# Perceptions of a Specific Family Communication Application among Grandparents and Grandchildren: An Extension of the Technology Acceptance Model

**DOI:** 10.1371/journal.pone.0156680

**Published:** 2016-06-07

**Authors:** Tsai-Hsuan Tsai, Hsien-Tsung Chang, Yi-Lun Ho

**Affiliations:** 1 Department of Industrial Design, Chang Gung University, Taoyuan, Taiwan; 2 Department of Computer Science and Information Engineering, Chang Gung University, Taoyuan, Taiwan; University of Perugia, ITALY

## Abstract

Many studies have noted that the use of social networks sites (SNSs) can enhance social interaction among the elderly and that the motivation for the elderly to use SNSs is to keep in contact with remote friends and family or the younger generation. Memotree is designed to promote intergenerational family communication. The system incorporates the Family Tree design concept and provides family communication mechanisms based on the Family Communication Scale. In addition, the system optimizes hardware and interface use to conform to the specific needs of older and substantially younger individuals. Regarding the impact of variables on SNS with respect to the interaction of usability variables in the construction of a cross-generational communication platform, we adopted the TAM model and Chung et al.’s suggestions to promote user acceptance of the proposed Memotree system. A total of 39 grandchildren and 39 grandparents met the criteria and were included in the study. The elderly and young respondents revealed substantial willingness to use and/or satisfaction with using the Memotree system. Empirical results indicate that technology affordances and perceived ease of use have a positive impact on perceived usefulness, while perceived ease of use is affected by technology affordances. Internet self-efficacy and perceived usefulness have a positive impact on the user’s behavioral intention toward the system. In addition, this study investigated age as a moderating variable in the model. The results indicate that grandchildren have a larger significant effect on the path between perceived usefulness and behavioral intention than grandparents. This study proposes a more complete framework for investigating the user’s behavioral intention and provides a more appropriate explanation of related services for cross-generational interaction with SNS services.

## Introduction

The aging of the global population is changing life and marriage patterns as well as population structures [1]. As life expectancy increases, more time is available for the development of family relationships, and elderly individuals are willing to spend more time on interaction with family members and maintaining family relationships [2]. Most grandparents attach considerable importance to their relationships with their grandchildren and are eager for more frequent and intimate interaction with them [3]. From the standpoint of grandparents, through this unilateral, passive acceptance of family assistance and support, the grandparents provide their grandchildren with material and emotional assistance. Such behavior satisfies and affirms the self-worth of the grandparents, increases interaction and improves emotional ties between the generations. It can also improve the vitality of the grandparents while slowing the onset of mental or physical illness [4]. In addition, grandparents have many years of experience to pass down to the younger generation, which provides their grandchildren with an understanding of life’s continuity and feelings of affirmation and respect. Affirmation and respect gained in this manner can raise the status and authority of the grandparents in the family, thus enhancing their self-identity and social value [4–9]. Interaction between grandchildren and their grandparents can enhance mutual understanding, reduce prejudice and fear of the elderly, and increase awareness of the nature of aging among the younger generation. In addition, through the learning process, the grandparents contribute to the development of personal traits, literacy, social acclimation, and the physical and mental health of their grandchildren [4, 8]. In Chinese families, such close relationships between grandparents and grandchildren are common primarily due to the influence of filial piety as a longstanding ethical core of Chinese society. Filial piety is a Confucian idea that encompasses a broad range of behaviors, including children’s respect, obedience, loyalty, material provision, and physical care for parents. Thus, grown children are considered responsible for the care of their parents and obliged to have their aging parents live with them. It is common for three generations to live under one roof while providing mutual assistance. This arrangement contributes to close relationships between grandparents and grandchildren. However, as a 2010 report by the United Nations notes [10], this traditional arrangement is gradually fading with changes to social and economic structures, and since the end of the Second World War, the number of elderly individuals who live alone has sharply increased. This trend is also visible in other Asian countries, where rapid industrialization and modernization have been widely accompanied by urbanization and the concentration of jobs in particular areas. As the younger generations migrate to urban centers to seek employment, they are separated from their elderly parents, which limits the interaction between grandparents and grandchildren and decreases the frequency of contact. These dramatic social changes are having a significant impact on family structures, functions, and resources as well as family relations. The predominant model of traditional family organization has been gradually replaced by single-family or nuclear-family patterns. These changes to traditional living patterns have decreased the frequency of contact between grandparents and grandchildren, thus reducing the degree of mutual understanding and making interaction between generations a critical problem for modern family relationships. [7, 11–15]

The development of social technologies has changed modes of social interaction. Communication between grandparents and grandchildren is no longer confined to face-to-face interaction, with social technologies offering mechanisms and opportunities for increased contact and understanding through social networking sites (SNSs), such as Facebook and Twitter [16]. A study by the Pew Research Center on the use of SNSs demonstrates that such sites are widely used among individuals below the age of 40 years, with increased usage intensity among younger groups [17]. Brandtzæg and Heim (18) emphasized that the motivation for the use of SNSs is to maintain and promote social relationships, including keeping in touch with friends, participating in social activities and making new friends. The conventional wisdom holds that older individuals and grandparents are insulated from digital technology. However, this is not the case. A survey released in April 2012 revealed that 53% of Americans over the age of 65 years use the Internet or email [19]. Additionally, similar to younger people, older individuals experience life transitions and changes in their social scope. For instance, as they transition from high school to college, younger individuals rely on SNSs to link their past and present friendships. This practice is also true of the elderly [20]. The Pew report noted that because the elderly population has increased in recent years the number of individuals over 65 years of age who use SNSs has significantly increased. For example, in April 2009, 13% of elderly Internet users used SNSs. However, by May 2011, this number grew to 33%, an increase of 150% in approximately two years. In addition, the report noted that 34% of elderly Internet users have Facebook accounts, and 18% use SNSs daily [19]. Furthermore, many studies have noted that the use of SNSs or other web-based technologies, such as Skype or blogs, can enhance social interaction among the elderly and that the primary motivator among the elderly to learn to use such tools is the desire to maintain contact with remote friends and family or the younger generation [21–26]. Therefore, SNSs represent a means to maintain interaction between generations within a family and extend the social interaction of elderly users. However, younger users have transitioned from the use of Internet relay chats with friends to the use of SNSs, and they rarely use these tools to interact with older individuals [16, 20]. From the perspective of social function, current SNSs (e.g., Facebook, Twitter) are useful for promoting social interaction, but only among peers, friends and colleagues [18, 27–29], and the researchers have been unable to determine how SNS functionality can be best used to help maintain and promote relationships within families, thus improving interaction between grandparents and grandchildren.

**Fig 1:**
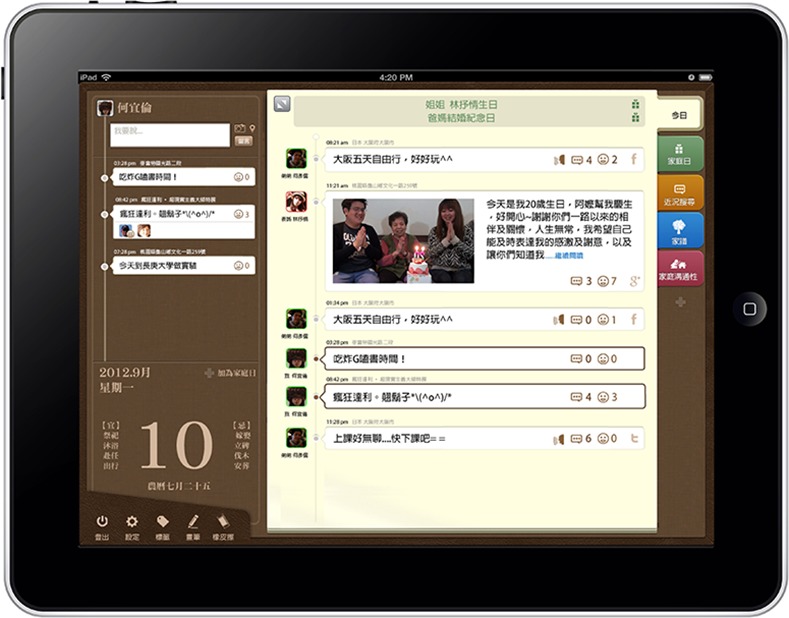
Memotree main page.

Several web-based communities have been developed in response to this increasing popularity of SNSs and the need for enhanced family interaction. For example, Family Tree is a Facebook-based application that enables the user to create a space for his or her family. In this space, the user can create and maintain a family-tree diagram, family photo albums, a record of family activities, and links to external family photo albums [30]. Famiva enables users and their family members to create a family tree, share family events and photographs, and develop a family map [31]. Geni.com focuses on family links and enables users to create a family tree and invite other family members to make entries in a family diary, edit the home timeline and share photographs [32]. eFamily is an application available on the Internet and mobile devices that enables users to share photographs and videos and create family photo albums and a family address book [33]. FamilyWall is another application that provides a private space for family members to communicate, organize family events, and share photographs and locations [34]. MyHeritage.com provides tools with which users can keep in contact with family members, celebrate birthdays, share photographs, and investigate and preserve their family history. First, the user creates a page for his or her family and then invites other family members to join [35]. However, such special-purpose SNSs do not enjoy the same level of popularity as general-purpose SNSs, such as Facebook and Twitter. In addition to having to set up a new account and establish a new social network, users must take time to familiarize themselves with the functions and interfaces of each of these services. In addition, the websites may not be suitable for elderly users for four main reasons. (1) Inappropriate hardware operation: The text size and fonts used in the interfaces present visual challenges for elderly users, and such users also have difficulty with mouse-based input (e.g., scroll-wheel operation and the relative positioning of mouse and cursor). In addition, status updates, messaging and chat features require typing, which represents a challenge for many older individuals [36–39]. (2) Lack of familiarity with Internet jargon: Terms that younger individuals take for granted on SNSs (e.g., photo upload, account, inbox) are unfamiliar to older individuals, which makes it difficult for them to successfully navigate and use the provided services [40]. (3) Overly complex operational processes: The extensive use of icons and navigation processes can make it difficult for elderly users to effectively use such sites, particularly in combination with unfamiliar terms [41–44]. (4) Inappropriate intergenerational family interaction: Today’s SNSs do not offer interfaces appropriate for use by older grandparents and younger grandchildren in terms of their intergenerational social habits and modes of interaction or links to the SNSs that they currently use [44–47]. For these reasons, this study describes the construction of a communication system, known as Memotree, that is designed to promote intergenerational family communication. As shown in Fig 1, Memotree not only provides functions similar to other SNSs, such as tools to diagram and track one’s family tree, family history and family organization, but also provides family communication mechanisms designed in accordance with the Family Communication Scale. Additionally, the system optimizes hardware and interface use to conform to the specific needs of older and much younger individuals. However, acceptance of the Memotree platform and its services by its target users and Memotree’s impact on family communication require investigation.

In the field of communications technology research, intention and attitude are key issues. The Technology Acceptance Model (TAM) developed by Davis et al. has established a favorable reputation among theorists as a tool to assess and predict user acceptance of information systems (IS) and information technology (IT). The Technology Acceptance Model was first proposed by Davis et al. to assess user acceptance of new technologies. The model was based on the theory of reasoned action [3] and the theory of planned behavior (TPB). The TAM consists of six variables: perceived usefulness (PU) [17], perceived ease of use (PEOU), attitude toward using the technology (A), behavioral intention to use (BI), actual system use and external variables. To thoroughly define determinants that affect PU, PEOU and BI, Venkatesh and Davis have continuously refined the TAM and proposed the extension of the Technology Acceptance Model (TAM2) [48], the unified theory of the acceptance and use of technology (UTAUT) [49] and the Technology Acceptance Model 3 (TAM3) [50]. All of the previously mentioned models are based on the user perspective to investigate user acceptance of a technological system and effectively predict and explain user motivation and behavior. In recent years, TAM has also been expanded to investigate the behavior patterns and intentions of SNS users. For example, TAM has been used to examine factors that affect the SNS usage intention among Malaysian students [51], to analyze user behavior in operating SNSs [52] and to investigate the behavioral intentions of Facebook users [53]. Constantinides et al. [54] developed and validated TAM to analyze the impact of various factors on SNS users in the Netherlands. Dhume et al. [55] applied TAM to SNS use among business-school students. Brandyberry et al. [56] used TAM to study behavioral intention for SNS users. Yang et al. [57] applied TAM to study the willingness of Taiwanese manufacturing staff members to use SNSs as learning aids, and Hu et al. [58] applied TAM to study the intention of non-SNS users to join SNSs. In addition, Chung et al. [59] suggested that TAM is applicable for in-depth investigation of online groups, proposed extending TAM by including age as a moderator and tested its validity in an online application. These studies demonstrate that TAM is suitable for the investigation and assessment of SNS user behavior and intention. Given that Memotree is a community-based website, this study adopts TAM and the suggestion of Chung et al. [59] that TAM can be extended to assess user acceptance of the proposed Memotree family communication system, including the willingness of grandchildren and grandparents to use the system and the satisfaction they derive from it.

## Hypothesis Development

**Fig 2:**
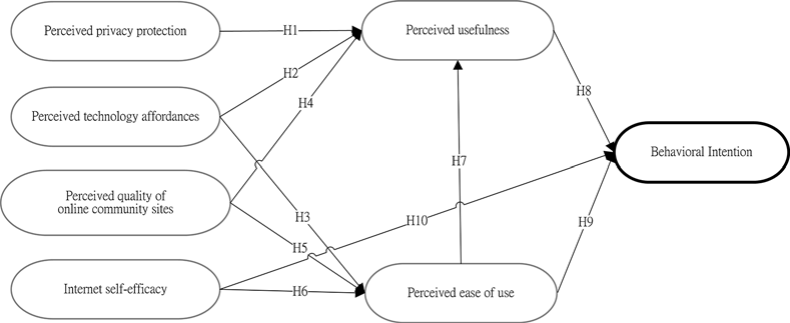
Memotree Research Structure.

This study aims to verify user acceptance of the Memotree communication system by understanding the underlying factors that influence the intention to use and the satisfaction of grandparent/grandchild users. Based on the original TAM, Chung et al. [59] proposed an extension of the technology acceptance model to assess an online community. The present study adopts Chung et al.’s [59] proposed external variables (i.e., Internet self-efficacy, quality of online community sites, technology affordances and privacy protection) to investigate the intention to adopt the Memotree system among potential grandparent/grandchild users. Additionally, TAM is derived from TRA, and according to TRA, a person's performance of a specified behavior is determined by the person’s behavioral intention (BI). BI is also jointly determined by the person's attitude (A) and subjective norm (SN) concerning the behavior in question. Any other factors that influence behavior do so only indirectly by influencing A, and SN would fall into the category “external variable.” Thus, the four variables proposed by Chung et al. [59] are termed “external variables.” Operational definitions of the variables are provided in Table 1, while the study’s research framework is shown in Fig 2.

**Table 1:** Operational definitions.

Variable	Operational definition
Perceived privacy protection	Degree to which Memotree protects user data
Perceived technology affordances	Degree to which Memotree provides users with valuable social functions
Perceived quality of online community sites	Degree to which users approve of the Memotree system and interface
Internet self-efficacy	Degree to which users feel confident completing a variety of online actions
Perceived usefulness	Degree to which users believe Memotree can improve family communication
Perceived ease of use	Degree to which users find Memotree tasks easy to learn and complete
Behavioral intention	Subjective probability of users using the Memotree system for family communication

According to Chung et al.’s [59] extension of the technology acceptance model, attitude toward using (A) was excluded from the original TAM, and perceived usefulness (PU), perceived ease of use (PEOU) and behavioral intention to use (BI) were retained [17]. In addition, four external variables were added to investigate their effect on PU and PEOU: Internet self-efficacy, perceived quality, technology affordances and privacy. First, Chung et al. [59] suggest that Internet self-efficacy has a significant impact on perceived ease of use and that the quality of online community sites can increase the frequency of user visits and strengthen the degree of participation. According to social cognitive theory (SCT), self-efficacy is related to one’s judgment of acquired skills rather than the actual obtained skills, i.e., the belief one has in one’s ability to complete a task in a particular situation. In a study on the Internet that investigated the basic operation of specific software and systems, computer/Internet self-efficacy is defined as one’s personal faith and confidence with respect to accomplishing a related task in the Internet environment, to organizing and executing appropriate action, to personally achieving a goal and to being able to use the skill in the future. In addition, Internet self-efficacy is a direct or indirect factor that affects the user’s ability to use and acceptance of a new technology [60, 61]. Related research, such as Igbaria and Iivari [62], used computer self-efficacy as a predicted variable in a questionnaire-based investigation. Their results indicated that computer self-efficacy has no direct effect on the willingness to use a computer but has a negative relation with computer anxiety and a positive relation with computer perceived ease of use. Venkatesh and Davis [63] also proposed that computer self-efficacy and objective usability have a significant effect on perceived ease of use. Recent TAM research on social network websites found that when users have a higher sense of Internet self-efficacy when operating a computer, this sense has a positive effect on the perceived ease of use of SNS [57, 59, 64]. Additionally, Gangadharbatla’s study [65] on the impact of Internet self-efficacy, the need to belong, the need for cognition, and collective self-esteem on the attitude toward SNS found that Internet self-efficacy, the need to belong and collective self-esteem have direct positive effects on the attitude toward SNS. Second, regarding the perceived quality of online community sites, Chung et al. [59] indicate that the quality of online community sites is positively associated with perceived ease of use and perceived usefulness. Noh and Lee [66] examine the relationship between quality factors and intention to use within the mobile application environment and find that improved quality in mobile applications in terms of information and service quality can enhance the intention to engage in mobile application-based services. Third, as demonstrated by Chung et al. [59], SNSs must provide users with unique and valuable social functions, and the user’s degree of technology affordances directly affects his/her perceived ease of use and perceived usefulness. Last, privacy has been a significant issue for SNS users. Individuals who use Facebook, Twitter, and other social-networking sites have expressed concerns regarding privacy, such as whether personal information can be disclosed, past posts are logged, profiles are deleted, and whether highly personal information can be disclosed in response to a warrant. Research has demonstrated that the perceived risk involved in uploading information to community websites has a significant impact on the willingness of non-community members to use such a site [58]. In addition, privacy protection is viewed as having a significant impact on perceived usefulness for SNS users [59, 67]. This study adopts Chung et al.’s [59] proposed external variables (i.e., Internet self-efficacy, quality of online community sites, technology affordances and privacy protection) to investigate the intention to adopt the Memotree system among potential grandparent/grandchild users. In addition, the relationships among perceived ease of use, perceived usefulness, technology affordances, and intention to participate in online communities are investigated.

Based on the previously outlined framework and the definitions of the operational variables, the following hypotheses are proposed:

H1 Perceived privacy protection will be positively associated with perceived usefulness of the Memotree system.
H2 Perceived technology affordances will be positively associated with perceived usefulness of the Memotree system.
H3 Perceived technology affordances will be positively associated with perceived ease of use of the Memotree system.
H4 Perceived quality of online community sites will be positively associated with perceived usefulness of the Memotree system.
H5 Perceived quality of online community sites will be positively associated with perceived ease of use of the Memotree system.
H6 Internet self-efficacy will be positively associated with perceived ease of use of the Memotree system.
H7 Perceived ease of use will be positively associated with perceived usefulness of Memotree system.
H8 Perceived usefulness will be positively associated with behavioral intention among Memotree users.
H9 Perceived ease of use will be positively associated with behavioral intention among Memotree users.
H10 Internet self-efficacy will be positively associated with behavioral intention among Memotree users.

## Methods

### Sampling and survey administration

Elderly grandparents and younger grandchildren were recruited as participants in the study. In terms of subject selection, the participants had to meet the planned screening criteria of the research proposal, which included the following factors: (a) being a grandparent; (b) having at least one grandchild aged 16 to 30 years; (c) having the ability to read and type; (d) having no significant cognitive, visual, hearing impairments, or medical conditions that render the participant unable to participate or use the system based on the Short Portable Mental Status Questionnaire (SPMSQ) screening result [68]; and (e) having the ability to participate in the experiments with a grandchild. We obtained ethical approval for our study from the Institutional Review Board (IRB), Chang Gung Hospital, Taoyuan, Taiwan (102-0840D). All relevant ethical safeguards have been met in relation to ethical consideration and subject protection. After we carefully explained the study’s purpose, the subjects were required to correctly orally answer 10 questions in the SPMSQ to verify their mental and cognitive state. All subjects signed informed consent forms before participating. In addition, the subjects of the photographs that appear in this manuscript provided written informed consent to the publication of their photographs.

**Fig 3:**
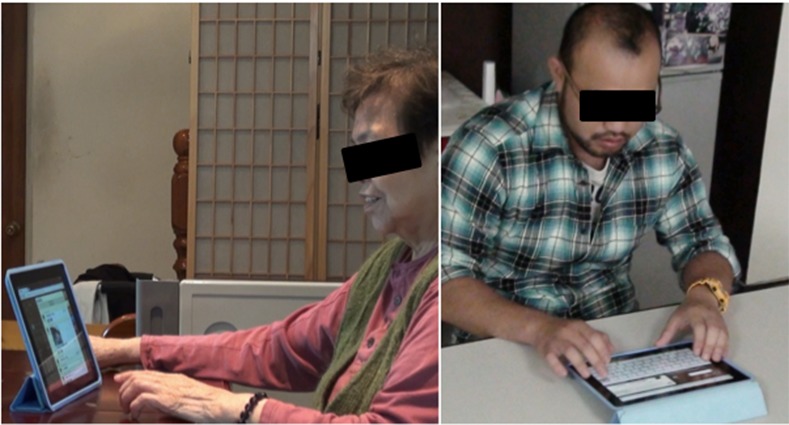
Demonstration of the interactive use of Memotree by the grandparent and grandchild.

Initially, a total of 42 grandchildren and 42 grandparents were recruited. However, during pre-analysis data screening, three grandparents were detected as unengaged respondents. Therefore, three grandparents and their grandchildren’s data were deleted, which left us with 39 grandchildren and 39 grandparents. Of the grandchildren respondents, 48.7% were female and 51.3% were male, with an average age of 24.4 years. Of the grandparent respondents, 69.2% were female and 30.8% were male, with an average age of 69.5 years. All grandchildren had previous experience using SNSs, while only eight grandparents had similar experience. Table **2** and Table 3 show the detailed sample demographics. To increase the convenience for the subjects, testing was conducted in the respondent’s home, in nearby community centers, and in the communal lounge in the New Taipei City Senior Fellows Association building. The grandchildren respondents were tested at the Chang Gung University Digital Media Lab (Fig 3). The study observed the operation of the Memotree family communication system by participants of different generations. All subjects completed the required tasks and a technology acceptance questionnaire. To improve the reliability and validity of the test, each test process and procedure was conducted in accordance with a set of standard operating procedures. Prior to beginning the questionnaire, the researcher informed the subject of the test purposes, outlined the test process and introduced the equipment used in the test. Each test session required approximately 30 minutes, during which the participant used the Memotree system on a tablet computer. Once the tasks were complete, the participant was asked to orally answer a questionnaire designed to measure the degree of technology acceptance while the experimenter recorded the data on a notebook computer Excel sheet. The testing stage was audio- and video-recorded to serve as a reference during analysis.

**Table 2:** Descriptive statistics of young respondent characteristics.

Gender	N	%	Age	N	%	Education	N	%	SNS Experience	N	%
Female	19	48.7	20–24	30	76.9	University	14	35.9	Yes	39	100.0
Male	20	51.3	25–29	8	20.5	Master	25	64.1	No	0	0.0
Total	39	100.0	30–34	1	2.6	Total	39	100.0			
			Total	39	100.0						

**Table 3:** Descriptive statistics of older respondent characteristics.

Gender	N	%	Age	N	%	Education	N	%	SNS Experience	N	%
Female	27	69.2	60~64	6	15.3	University	12	30.7	Yes	8	20.5
Male	12	30.8	65~69	17	43.5	Master	1	3.5	No	31	79.5
Total	39	100.0	70~74	11	28.2	High school	14	35.8	Total	39	100
			75~79	3	7.6	Junior high school	3	7.7			
			85~89	2	5.4	Elementary school	9	22.3			
			Total	39	100.0	Total	39	100.0			

### Material

The Memotree user interface was designed using the Notepad concept and emphasizes interaction between family members and event recording. In addition, the system links directly to other current SNSs, including Facebook and Google+, and adopts design and operation features that are familiar to users of such platforms. Memotree is designed to support a range of hardware configurations, including conventional PCs and smart mobile devices, such as tablet PCs, thus providing flexibility and accessibility for elderly users who may not be familiar with computers. The main design concept of Memotree is as follows:

(1) Family Tree design concept

**Fig 4:**
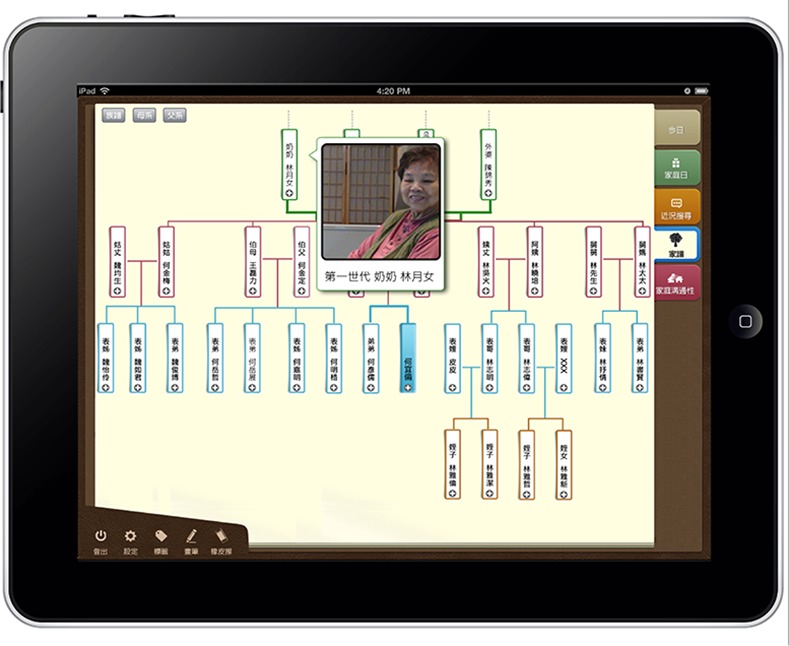
Family tree.

The Memotree system includes a Family Tree feature, which enables the family members to diagram their family tree and record their family’s lineage and family relationships. The user can manually manage his or her family tree, adding or deleting members, and use auxiliary functions, such as the calendar, to record family celebrations, birthdays and anniversaries. Family Tree emphasizes relationships between the current three generations of the family, and Memotree focuses on locating the user within those relationships, with the older and younger generations displayed above and below, respectively (Fig 4).

(2) Family Communication component function design

**Fig 5:**
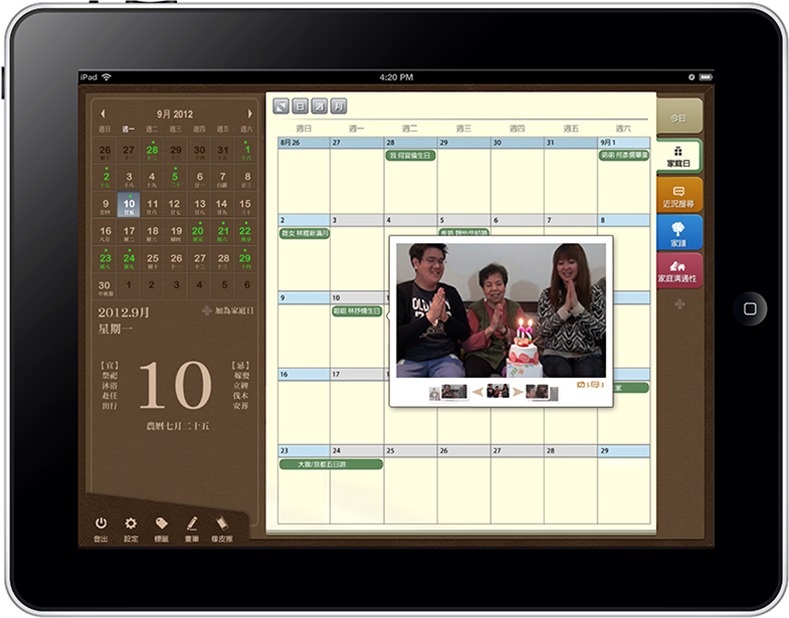
Memotree family calendar.

To promote communication and interaction among family members, the Olson Family Communication Scale (FCS) was used to design the Memotree functions. This scale uses skills, including listening, speaking, self-disclosure, tracking, and respect and regard, to assess the degree of communication among family members [69, 70]. Memotree’s video and chat rooms promote listening and speaking among family members by enabling them to confidentially orally share their thoughts while looking at one another because the use of video simulates genuine face-to-face interaction. Memotree status updates and photo-upload features help meet the self-disclosure requirements, with status updates enabling family members to confidentially share thoughts, feelings and recent events with family members. Memotree’s Self-disclosure space provides users a personalized, open forum in which to express their feelings and ideas, which enables family members to learn more about one another. The Tracking feature enables family members to discuss various topics using text and emoticons, which provides them emotional support and encouragement. Memotree also enables users to group messages and responses by theme for storage in the Family Calendar System (Fig 5), which enables all family members to search the calendar to place events and family activities in the correct context. In terms of the respect-and-regard family communication functions, Memotree’s Family Calendar System records important family events, such as birthdays, memorials, anniversaries and other important days. Memotree users can manually set their own Family Day. In addition, the Family Calendar reminds users and their family members of family celebrations and provides a venue in which to express blessings, respects and best wishes.

(3) Personalized Family Communication assessment and reminder mechanism

**Fig 6:**
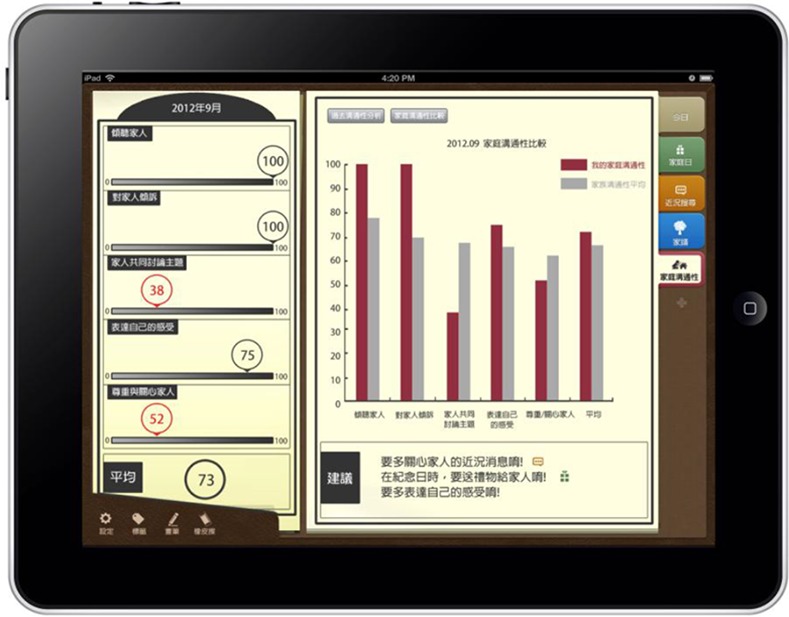
Family communication measure.

Using the Olson Family Communication Scale as the basis for evaluation, the Memotree system compiles statistics on function use by each family member over the previous month to assess family communication. In Fig 6, the left column shows the user’s family communication score, which ranges from 0 to 100. A number that appears in red indicates a lack of communication skills and interaction among family members. Regarding the right column in Fig 6, two options are provided: past communication skills analysis and comparison of communication skills. Past communication skill analysis reveals the communication skill performance history within a family, while comparison of communication skill indicates the communication relationship between the user and other family members. A histogram expresses the comparison result between the user and other family members. Red bars indicate the user’s latest communication skill score in the family, while gray bars indicate the communication skills score of other family members. Family members can access these data through the system, which can help them obtain a better understanding of the degree of communication within the family, and provides appropriate suggestions and reminders to help family members maintain good communication.

### Measurement development and statistical analysis

The survey instrument was based on the survey developed by Chung et al. [59], with modifications to match the research topic and to eliminate irrelevant items. The survey asked participants to respond to a series of statements using a 5-point Likert-type scale (1 = strongly disagree to 5 = strongly agree). In accordance with the research goals and considering the appropriateness of measurement scales and statistical analysis tools, this study adopted technology acceptance tests as the basis for analysis and overall pattern analysis.

During the testing period, questionnaire responses were processed continuously. The questionnaires were first reviewed to ensure completeness and validity, thus preventing data-input errors. Each completed questionnaire was assigned a serial number and then input into an SPSS data file. When the valid questionnaires were completely inputted, they were reviewed again to correct input errors. Section 4.1 provides descriptive statistics for each TAM question. Next, the research hypotheses were evaluated using structural equation modeling (SEM). SEM is a powerful multivariate technique that facilitates specification of the relationships between and among variables (measured variables and latent constructs) using two components of a causal model: a measurement model and a structural model. Fig 2 represents the structural model being examined. The structural model describes the hypothesized relationships among theoretical constructs. For each construct in Fig 2, there was a single measurement model. The model links the construct in the diagram with a set of variables, which consist of the relationships between the latent variables and their measures, presented as the part of the model. In SEM, the measurement model is estimated using confirmatory factor analysis (CFA) to test whether the constructs demonstrate sufficient reliability and validity. In this study, SPSS and AMOS 20.0 were used to test the structural equation model. Sections 4.2 and 4.3 describe the analysis results of the measurement model and the test of the structural model, respectively. Subsequently, to understand how generational differences affect the use of the Memotree system, in Section 4.4, we used ANOVA to analyze age difference-based variation among Memotree users.

## Results

### Descriptive statistics

To verify that each questionnaire item was statistically significant, first, descriptive statistics were applied for each question. The results are presented in Table 4, in which the descriptive statistics section reveals that each question scored on average between 3.9487 and 4.4821 and that the standard deviation for each question was between 0.585 and 0.784. On average, users responded positively to the Memotree system, and except for perceived privacy protection, which was near four, the averages exceeded four out of five. The participants responded positively to all seven constructs (perceived privacy protection, perceived technology affordances, perceived quality of online community sites, Internet self-efficacy, perceived usefulness, perceived ease of use, and behavioral intention) in using Memotree operations. This outcome indicates that TAM in this study tends toward accepting the use of the family communication platform.

**Table 4:** Descriptive statistics of TAM items (n = 78).

Constructs	Mean	Standard Deviation
Perceived privacy protection	3.9487	0.784
Perceived technology affordances	4.4821	0.585
Perceived quality of online community sites	4.3205	0.650
Internet self-efficacy	4.4444	0.647
Perceived usefulness	4.3974	0.601
Perceived ease of use	4.2222	0.711
Behavioral intention	4.1452	0.720

### Measurement model

For the analysis of the measurement model, we first obtained the Cronbach’s alpha coefficient to measure the reliability and validity of the questionnaire’s constructs. The goal of using Cronbach’s alpha was to test the reliability of the individual items with respect to the corresponding construct variable. The Cronbach’s alpha values for all items were over 0.7, and the item-total correlation for each item exceeded 0.3, thus satisfying the research criteria.

Table 5 shows that the Cronbach’s alpha result fulfills the criteria for reliability for the following variables: perceived ease of use, Internet self-efficacy, perceived usefulness, behavioral intention, perceived quality of online community sites, perceived technology affordances and perceived privacy protection. Nearly all of the relevant coefficients for the individual and item-total correlation for each dimension were over 0.4, which indicates that the instrument has a high degree of reliability and the measured results can be considered highly stable. Factor loading was also obtained by conducting confirmatory factor analysis (Table 5). Factor loading refers to how much a factor (or item in our study) explains a variable. According to Hair et al. [71], with a sample size of 85, the factor loading should be at least 0.60 to consider a factor reliable. In our research, the factor loading of each item surpassed the threshold.

**Table 5:** Reliability and validity of measurement model.

Variable	Item	Item reliability (Cronbach's α value)	Factor loading	Composite reliability	Average variance extracted	Item-total correlation
Perceived usefulness	PU1	0.9099	0.800	0.923	0.801	0.4998
	PU2	0.9075	0.932			0.6396
	PU3	0.9070	0.946			0.6647
Perceived ease of use	EOU1	0.9073	0.852	0.905	0.760	0.6209
	EOU2	0.9079	0.833			0.5937
	EOU3	0.9067	0.928			0.6409
Behavioral intention	BI1	0.9085	0.908	0.932	0.821	0.5670
	BI2	0.9098	0.907			0.5058
	BI3	0.9094	0.903			0.5237
Internet self-efficacy	N1	0.9124	0.813	0.843	0.641	0.3660
	N2	0.9114	0.833			0.4237
	N3	0.9136	0.928			0.3068
Perceived quality of online community sites	Q1	0.9096	0.868	0.882	0.715	0.5136
	Q2	0.9095	0.805			0.5191
	Q3	0.9091	0.862			0.5406
Perceived technology affordances	F1	0.9100	0.778	0.885	0.608	0.5039
	F2	0.9102	0.801			0.4930
	F3	0.9094	0.680			0.5219
	F4	0.9065	0.876			0.6811
	F5	0.9081	0.750			0.5966
Perceived privacy protection	P1	0.9103	0.897	0.944	0.849	0.5005
	P2	0.9076	0.949			0.6054
	P3	0.9088	0.917			0.5542

Then, we used convergent validity and discriminant validity to conduct efficiency analysis. Convergent validity refers to the extent to which the measured variables converge with the same construct. Sufficient convergent validity requires that the average variance extracted (AVE) of latent variables be no less than 0.5, and the latent variable composite reliability (CR) value should be greater than 0.7. Composite reliability is a measure of the overall reliability of a collection of heterogeneous but similar individual items. Table 5 shows that the AVE of the constructs used in this study is over the threshold value of 0.5 and the latent variable composite reliability values are over 0.8, which indicates that each construct in the measurement tool has good convergent validity. In addition, a measurement model should be capable of providing discriminant validity, whereby the square root of the AVE of the potential change items must be larger than the correlation coefficient of the other dimensions. Table **6** provides a matrix of the correlation coefficients (the figures above the diagonal are the square roots of the AVE). The table shows that the square root of the average change of the latent variables is larger than the correlation coefficient of the other dimensions, thus confirming the good discriminant validity of the constructs used in this study. In sum, the measurement model test, including convergent validity and discriminant validity, was satisfactory.

**Table 6:** Discriminant validity of the latent constructs.

	Behavioral intention	Perceived ease of use	Internet self-efficacy	Perceived privacy protection	Perceived quality of online community sites	Perceived technology affordances	Perceived usefulness
Behavioral intention	0.906						
Perceived ease of use	.372**	0.872					
Internet self-efficacy	.405**	.334**	0.800				
Perceived privacy protection	.197**	.455**	.204**	0.921			
Perceived quality of online community sites	.330**	.415**	.238**	.431**	0.845		
Perceived technology affordances	.380**	.541**	.293**	.431**	.494**	0.780	
Perceived usefulness	.454**	.567**	.186*	.414**	.393**	.529**	0.895

Note: (a) The diagonals represent the average variance extracted (AVE), while the other matrix entries represent the shared variance (the squared correlations). (b)

** P<0.01

* P<0.05.

Table **7** shows the model fit measures for the measurement model. Hair et al. [71] indicated that seven common fit indices can be used to test the measurement model fit: chi-square/ degree of freedom (X^2^/df), goodness-of-fit index (GFI), adjusted goodness-of-fit index (AGFI), normalized fit index [20], non-normalized fit index (NNFI), comparative fit index (CFI), and root mean square error of approximation (RESEA). In terms of the fit indices of the measurement model, the X^2^/df value is 1.875, which meets the value threshold of less than 3.0. The remaining indices failed to meet the threshold. The GFI and AGFI values were 0.734 and 0.649, respectively. These values should be larger than 0.8 to be considered a good model fit. The NFI, NNFI and CFI values were 0.717, 0.864 and 0.838, respectively. These values should be larger than 0.9 to be considered a good model fit. The RMSEA value was 0.107. This value should be less than 0.08 to be considered a good model fit. After combining the results, the measurement model’s fit index values were unsatisfactory. We assume the subject sampling size affected the fit indices for the measurement and structural models. Research has demonstrated that certain fit indices are sensitive to sample size. For example, Sharma et al. [72] stated that GFI and AGFI are affected by sample size, while other studies claim that when samples are small, NFI is often underestimated [73–75].

**Table 7:** Fit indices for the measurement model.

Measures	Recommended criteria	Suggested by authors	Measurement model
χ2/df	< 3.0	Bentler and Bonett (76)	1.875
GFI	> 0.8	Seyal, Rahman (77)	0.734
AGFI	> 0.8	Scott (78)	0.649
NFI	> 0.9	Hair Jr, Anderson (79)	0.717
NNFI	> 0.9	Hair Jr, Anderson (79)	0.864
CFI	> 0.9	Bagozzi and Yi (80)	0.838
RMSEA	< 0.08	Bagozzi and Yi (80)	0.107

### Structural model

**Fig 7:**
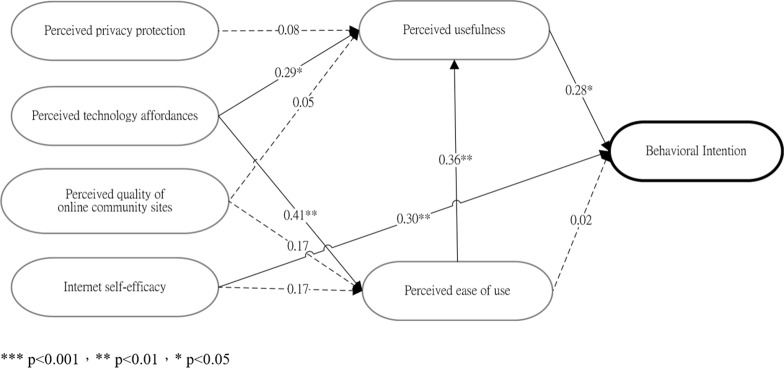
Path analysis model for research hypotheses.

We examined the structural equation model by testing the hypothesized relationship among the seven latent research variables: perceived ease of use, Internet self-efficacy, perceived usefulness, behavioral intention, perceived quality of online community sites, perceived technology affordances and perceived privacy protection. Fig 7 explains the verification results for the hypotheses. In the figure, bold lines indicate “significant” and dotted lines indicate “non-significant.”

Analysis results using the structural equation model indicate that not all of the study’s hypotheses are fully supported. Table **8** presents the path coefficients and statistical measurements. H1 holds that perceived privacy protection is positively correlated with perceived usefulness among users of the Memotree system. The analysis results indicate a path such that γ1 = 0.08(t = 0.77, p = 0.455), which fails to achieve significance. Thus, H1 is not supported. H2 holds that perceived technology affordances are positively correlated with perceived usefulness among Memotree users. The analysis results indicate a path such that γ2 = 0.29 (t = 2.51, p = 0.026), thus achieving a significance of p<0.05. Therefore, H2 is supported. H3 holds that perceived technology affordances are positively correlated with perceived ease of use among Memotree users. The analysis results indicate a path such that γ3 = 0.41 (t = 3.73, p = 0.002), thus achieving a significance of p<0.01. Therefore, H3 is supported. H4 holds that perceived quality of online community sites is positively correlated with perceived usefulness among Memotree users. The analysis results indicate a path such that γ4 = 0.05 (t = 0.47, p = 0.646), which fails to achieve significance. Thus, H4 is not supported. H5 holds that perceived quality of online community sites is positively correlated with perceived ease of use among Memotree users. The analysis results indicate a path such that γ5 = 0.17 (t = 1.56, p = 0.142), which fails to achieve significance. Thus, H5 is not supported. H6 holds that Internet self-efficacy is positively correlated with perceived ease of use among Memotree users. The analysis results indicate a path such that γ6 = 0.17 (t = 1.78, p = 0.098), which fails to achieve significance. Thus, H6 is not supported. H7 holds that perceived ease of use is positively correlated with perceived usefulness among Memotree users. The analysis results indicate a path such that γ7 = 0.36 (t = 3.35, p = 0.005), thus achieving a significance of p<0.01. Therefore, H7 is supported. H8 holds that perceived usefulness is positively correlated with behavioral intention among Memotree users. The analysis results indicate a path such that γ8 = 0.28 (t = 2.87, p = 0.013), thus achieving a significance of p< 0.05. Therefore, H8 is supported. H9 holds that perceived ease of use is positively correlated with behavioral intention among Memotree users. The analysis results indicate a path such that γ9 = 0.02 (t = 0.65, p = 0.527), which fails to achieve significance. Thus, H9 is not supported. H10 holds that Internet self-efficacy is positively correlated with behavioral intention among Memotree users. The analysis results indicate a path such that γ10 = 0.30 (t = 3.24, p = 0.006), thus achieving a significance of p<0.01. Therefore, H10 is supported.

**Table 8:** Summary of hypothesis tests.

Exogenous variable		Endogenous variable	Standardized regression coefficient	T-value	P-value	Support
H1. Perceived privacy protecttion	→	Perceived usefulness	0.08	0.77	0.455	No
H2. Perceived technology affordances	→	Perceived usefulness	0.29	2.51	0.026*	Yes
H3. Perceived technology affordances	→	Perceived ease of use	0.41	3.73	0.002**	Yes
H4. Perceived quality of online community sites	→	Perceived usefulness	0.05	0.47	0.646	No
H5. Perceived quality of online community sites	→	Perceived ease of use	0.17	1.56	0.142	No
H6. Internet self-efficacy	→	Perceived ease of use	0.17	1.78	0.098	No
H7. Perceived ease of use	→	Perceived usefulness	0.36	3.35	0.005**	Yes
H8. Perceived usefulness	→	Behavioral intention	0.28	2.87	0.013*	Yes
H9. Perceived ease of use	→	Behavioral intention	0.02	0.65	0.527	No
H10. Internet self-efficacy	→	Behavioral intention	0.30	3.24	0.006**	Yes

*** p<0.001

** p<0.01

* p<0.05.

Table **9** shows the fit indices for the structural model. The chosen fit indices were the same as the fit indices for the measurement model. In terms of the fit indices of the structural model, all values meet the threshold except for the RMSEA value, which was 0.081, thus failing to meet the recommended threshold 0.08. That is, the X^2^/df value was 1.49, which was less than the recommended threshold of 3.0. The GFI and AGFI values were 0.97 and 0.85, respectively. These values were larger than the 0.8 considered to represent a good model fit. The NFI, NNFI and CFI values were 0.97, 0.96 and 0.99, respectively. These values were larger than the recommended threshold value 0.9. The results indicate that the fit indices for the structural model were satisfactory.

**Table 9:** Fit indices for the structural model.

Measures	Recommended criteria	Suggested by authors	Structural model
χ2/df	< 3.0	Bentler and Bonett (76)	1.49
GFI	> 0.8	Seyal, Rahman (77)	0.97
AGFI	> 0.8	Scott (78)	0.85
NFI	> 0.9	Hair Jr, Anderson (79)	0.97
NNFI	> 0.9	Hair Jr, Anderson (79)	0.96
CFI	> 0.9	Bagozzi and Yi (80)	0.99
RMSEA	< 0.08	Bagozzi and Yi (80)	0.081

### Age difference analysis

In addition, this study uses age as a moderating factor to provide insight into the impact of elderly users on each variable and path coefficient. First, age is set as the variable factor for single-factor variable analysis. As shown in Table **10**, the results indicate that the F value of perceived ease of use is 4.426, P<0.05; perceived usefulness is 11.461, P<0.001; behavioral intention is 6.926, P<0.05; perceived quality of online community sites is 4.716, P<0.05, and perceived privacy protection is 7.996, P<0.05. Thus, these values are significant. It can be inferred that age has an impact on these variables. Second, age and the independent variables are multiplied to assess the moderating variables. When the regression coefficients are multiplied, age is found to have a moderating effect. That is, the path will be affected by age. As shown in Table **11**, age has a moderating effect (β = -1.58*, p< 0.05) on perceived usefulness and behavioral intention such that the impact of perceived usefulness on behavioral intention differs with age. Table 12 indicates that the relationship between perceived usefulness and behavioral intention was significant for grandchildren but not for grandparents.

**Table 10:** ANOVA analysis of age for each variable.

	Grandchildren mean value	Grandparent mean value	F	P-value	Support
Perceived ease of use	4.0769	4.3675	4.426	0.039	Yes
Perceived usefulness	4.2051	4.5897	11.461	0.001	Yes
Behavioral intention	3.9572	4.3333	6.926	0.010	Yes
Internet self-efficacy	4.4444	4.4444	0.000	1.000	No
Perceived quality of online community sites	4.1880	4.4529	4.716	0.033	Yes
Perceived technology affordances	4.4307	4.5333	1.048	0.309	No
Perceived privacy protection	3.7264	4.5333	7.996	0.006	Yes

**Table 11:** Moderating impact analysis of age.

Hypothesis	β	T-value	Support
Perceived privacy protection *age→perceived usefulness	0.053	0.82	No
Perceived quality of online community sites* age→perceived usefulness	0.011	0.16	No
Perceived technology affordances* age→perceived usefulness	0.073	1.19	No
Internet self-efficacy* age→perceived ease of use	0.087	1.21	No
Perceived technology affordances* age→perceived ease of use	0.087	1.21	No
Perceived quality of online community sites* age→perceived ease of use	0.069	0.93	No
Perceived ease of use* age→perceived usefulness	-0.048	-0.761	No
Perceived usefulness* age→behavioral intention	-1.58*	-1.98	Yes
Perceived ease of use* age→behavioral intention	-0.079	-1.02	No

*** p<0.001

** p<0.01

* p<0.05.

**Table 12:** Regression result of perceived usefulness on behavioral intention differentiated by age.

	β	T-value	p
Grandchildren–Perceived usefulness	.643	3.924	.000***
Grandparents–Perceived usefulness	.211	0.962	.342

*** p<0.001

** p<0.01

* p<0.05.

## Discussion

In this study, we developed a family communication platform known as Memotree for intergenerational users based on the different user habits and experience or elderly and young individuals. The Memotree system makes it easy for grandparents to adopt the younger generation’s social mode and improve interaction and communication with other family members. The primary aim of the study was to use Chung et al.’s [59] proposed extension of the technology acceptance model to verify user acceptance and investigate intention/satisfaction among the grandparents and grandchildren through the use of the Memotree system. The results of the study indicate that a path was established between perceived ease of use and perceived usefulness, which does not correspond with the findings of Chung et al. [59]. The reason for this outcome is that the target groups assessed by Chung et al. were familiar with the operation of online communities. In contrast, as a newly developed social interaction platform, Memotree assumes that users must learn to use the new technology. Thus, perceived ease of use is a key factor in perceived usefulness. However, the significant path found between perceived usefulness and behavioral intention agreed with Chung et al., which indicates that Memotree is more likely to be accepted when users consider it to be useful or effective in enhancing family communication.

This study examined other external variables that may affect the willingness of users to adopt Memotree. We did not find a path between Internet self-efficacy and perceived ease of use, which does not conform with Chung et al.’s findings [59]. However, a significant path was found between Internet self-efficacy and the behavioral intention to use Memotree, which corresponds to Gangadharbatla’s findings [65] and indicates that confidence in using Memotree would lead users to a positive behavioral intention toward using the system. This study also found that the perceived quality of online community sites has no effect on perceived ease of use and perceived usefulness. This result did not agree with previous studies, all of which demonstrated positive associations between perceived quality and both perceived ease of use and perceived usefulness [66, 81–84]. Previous studies were primarily conducted using desktop computers and their associated operating systems. In contrast, the Memotree operating interface is designed to be used through touch screens on tablet computers. Therefore, the user experience of the different operating interface and the fact that Memotree is a newly developed system might explain why no significant paths were found. In terms of the technology affordances variables, the study concludes that technology affordances directly affect perceived ease of use and perceived usefulness, which agrees with Chung et al.’s findings [59]. Based on SEM, both grandparents and grandchildren believed that the unique social function Memotree provided could afford new opportunities or potential benefits within a family, which led them to believe that the system was easy to learn and operate (ease of use) and could improve family communication (perceived usefulness). Privacy protection is viewed as having a significant impact on perceived usefulness [59, 67]. Compared with previous studies in which the users had experience using online communities or SNS, in this study on Memotree, both user groups were only required to operate the system in an experimental environment and thus did not have a chance to use it in real-life circumstances. Therefore, they lacked the experience to determine whether the system’s privacy protection would affect their willingness to use Memotree. Thus, the path for privacy protection to perceived usefulness was non-significant.

In addition, this study divided users into two groups (grandparents and grandchildren) to investigate the differences between users who belong to different generations. The results indicate that age has a significant effect on perceived ease of use, perceived usefulness, behavioral intention, perceived quality of online community sites and perceived privacy protection. As shown in Table **10**, the mean scores of the grandparents are larger than the mean scores of the grandchildren. The explanation of this result could be that grandparents are not as sensitive or familiar with information technology as their grandchildren. Therefore, grandparents tend to award higher scores. Regarding the grandchildren, because they have more experience with and knowledge of information technology than their grandparents, they tend to be stricter when evaluating the system. However, overall, the mean scores of the grandparents and the grandchildren for perceived ease of use, perceived usefulness and perceived quality of online community sites were all over 4. For behavioral intention and perceived privacy protection, the mean scores of the grandparents were also over 4 and the mean scores of the grandchildren were close to 4, which indicates that although there are significant differences among users of different ages in evaluating the system, both groups were satisfied with Memotree. In addition, in this study, age is an adjustable variable, which means that it affects perceived usefulness and behavioral intention. This result differs from that of Chung et al. [59] primarily because of the organization of online communities and the goals of their users. The elderly users examined in this study lacked experience using online communities and thus had a low intention to use SNSs. However, Memotree is primarily used as a family communication system, and previous research results indicate that one of the key motivations for elderly individuals to use IT products is the opportunity to interact with other family members. Therefore, the goals of promoting family interaction and communication directly enhance the intention of elderly users to use Memotree.

## Conclusion

Previous TAM studies on the acceptance of social networking sites are too numerous to list. However, few have focused on the impact of external variables on SNS usability primarily because most current SNSs lack a mature model for the presentation of content and user behavior and because relevant empirical research is lacking. Therefore, this study followed Chung et al.’s proposals regarding the impact of variables on SNS with respect to the interaction of usability variables in the construction of a cross-generational communication platform known as Memotree. Empirical results indicate that technology affordances and perceived ease of use have a positive impact on perceived usefulness, while perceived ease of use is affected by technology affordances. Perceived usefulness and Internet self-efficacy have a positive impact on the user’s behavioral intention toward the system. In addition, this study investigated age as a moderating variable in the model. The results indicate that age only affects the path between perceived usefulness and behavioral intention. Therefore, in future research, the effect of age on perceived usefulness and behavioral intention could be investigated via interviews to understand why elderly individuals and young adults have different attitudes regarding perceived usefulness and behavioral intention. The finding could serve as a guideline when designing systems for different user age groups.

This study also used the cross-generational communication platform to verify the impact of external variables on SNS users. Compared with traditional technology acceptance behavior theory, this study proposes a more complete framework for investigating user behavioral intention and provides a more appropriate explanation of related services for cross-generational interaction with SNS services. The grandchildren and grandparents who served as participants in this study were mostly urban residents, which limits generalizing the findings to other geographic regions and environments. Follow-up studies can expand the investigation to include a wider range of subjects and a larger, more diverse sampling pool, thus increasing the reliability and validity of the findings. In addition, because we tested user acceptance of the Memotree system in an on-site situation in this study, little was learned regarding user acceptance in real-life circumstances. Therefore, longitudinal studies could be conducted to investigate practical use by grandparents and grandchildren and potential problems using the system under real-life conditions. Finally, this study was based on quantitative survey results. Future studies may consider using in-depth interviews, observation or other qualitative techniques to improve our understanding of the behavioral intentions and feelings of SNS users.

## Supporting Information

S1 Appendix Short Portable Mental Status Questionnaire (SPMSQ). (PDF)

S1 Dataset MemoTree Raw Data 78 Subjects Sheet. (PDF)
