# Supplementary material for: Perceptions of a Specific Family Communication Application among Grandparents and Grandchildren: An Extension of the Technology Acceptance Model
Source: PLoS One. 2016 Jun 7;11(6):e0156680. doi: 10.1371/journal.pone.0156680 (PMC4896451; doi:10.1371/journal.pone.0156680)
Supplement: S1 Appendix — (PDF) [file pone.0156680.s001.pdf]

Appendix A. Short Portable Mental Status Questionnaire (SPMSQ)

Patient's Name:

Date:

Sex: Male / Female

Yrs of Education: Master / University / High school / Junior high school / Elementary school

Instructions: Ask questions 1 to 10 on this list and record all answers. (Ask question 4a only if the subject does not have a telephone.) All responses must be given without reference to calendar, newspaper, birth certificate, or other aid to memory. Record the total number of errors based on the answers to the 10 questions.

+	–	Questions	Instructions
		1. What is the date today?	– Correct only when the month, date, and year are all correct.
		2. What day of the week is it?	– Correct only when the day is correct.
		3. What is the name of this place?	– Correct if any of the description of the location is given. "My home," the correct city/town, or the correct name of the hospital/institution are all acceptable.
		4. (a)What is your telephone number?	– Correct when the number can be verified or the subject can repeat the same number at a later time in the interview.
		(b)What is your street address?	– Ask only if the subject does not have a telephone.
		5. How old are you?	– Correct when the stated age corresponds to the date of birth.
		6. When were you born?	– Correct only when the month, date, and year are correct.
		7. Who is the president of Taiwan now?	– Requires only the correct last name.
		8. Who was president just before him?	– Requires only the correct last name.
		9. What was your mother's maiden name?	– Needs no verification; it only requires a female first name plus a last name other than the subject's.
		10. Subtract 3 from 20 and keep subtracting 3 from each new number, all the way down.	– The entire series must be performed correctly to be scored as correct. Any error in the series—or an unwillingness to attempt the series—is scored as incorrect.

Total Number of Errors

0~2 errors = Intact Intellectual Functioning

3~4 errors = Mild Intellectual Impairment

5~7 errors = Moderate Intellectual Impairment

8~10 errors = Severe Intellectual Impairment
