# Supplementary material for: Perceptions of a Specific Family Communication Application among Grandparents and Grandchildren: An Extension of the Technology Acceptance Model
Source: PLoS One. 2016 Jun 7;11(6):e0156680. doi: 10.1371/journal.pone.0156680 (PMC4896451; doi:10.1371/journal.pone.0156680)
Supplement: S1 Dataset — (PDF) [file pone.0156680.s002.pdf]

編號	Gender	Age	Education Level	SMS Experience	N1	N2
YOUNG1	Male	24	Graduate school	Yes	4	3
YOUNG2	Female	24	Graduate school	Yes	4	4
YOUNG3	Female	24	Graduate school	Yes	5	4
YOUNG4	Male	24	Graduate school	Yes	5	5
YOUNG5	Female	25	Graduate school	Yes	4	5
YOUNG6	Female	25	College	Yes	5	4
YOUNG7	Female	24	Graduate school	Yes	4	4
YOUNG8	Female	24	Graduate school	Yes	5	5
YOUNG9	Male	24	Graduate school	Yes	4	5
YOUNG10	Male	24	Graduate school	Yes	5	5
YOUNG11	Male	24	Graduate school	Yes	5	5
YOUNG12	Female	30	Graduate school	Yes	5	5
YOUNG13	Male	25	Graduate school	Yes	5	4
YOUNG14	Female	24	Graduate school	Yes	3	4
YOUNG15	Female	25	Graduate school	Yes	4	4
YOUNG16	Male	25	Graduate school	Yes	4	4
YOUNG17	Female	24	Graduate school	Yes	5	5
YOUNG18	Male	24	Graduate school	Yes	4	5
YOUNG19	Female	24	College	Yes	5	5
YOUNG20	Female	24	College	Yes	3	2
YOUNG21	Male	25	Graduate school	Yes	3	5
YOUNG22	Female	24	Graduate school	Yes	4	4
YOUNG23	Male	24	Graduate school	Yes	4	3
YOUNG24	Male	24	Graduate school	Yes	4	4
YOUNG25	Female	24	Graduate school	Yes	5	5
YOUNG26	Female	24	Graduate school	Yes	5	5
YOUNG27	Male	24	College	Yes	5	5
YOUNG28	Male	25	Graduate school	Yes	5	5
YOUNG29	Male	24	College	Yes	5	4
YOUNG30	Male	25	Graduate school	Yes	5	4
YOUNG31	Male	24	College	Yes	3	4
YOUNG32	Female	24	College	Yes	4	4
YOUNG33	Male	24	College	Yes	5	5
YOUNG34	Female	24	College	Yes	5	5
YOUNG35	Female	24	College	Yes	5	5
YOUNG36	Male	24	College	Yes	5	5
YOUNG37	Female	24	College	Yes	5	4
YOUNG38	Male	24	College	Yes	4	4
YOUNG39	Male	24	College	Yes	5	5
OLD1	Female	63	Elementary	No	4	3
OLD2	Female	71	Elementary	No	5	4
OLD3	Female	70	Senior High	No	4	5
OLD4	Female	74	Junior High	Yes	5	4
OLD5	Male	72	College	No	5	5
OLD6	Male	69	Senior High	Yes	5	5
OLD7	Female	70	Senior High	Yes	5	5
OLD8	Female	67	College	No	4	4

OLD9	Female	68	Senior High	Yes	5	5
OLD10	Male	71	College	Yes	5	5
OLD11	Female	66	Graduate school	No	5	5
OLD12	Male	69	Elementary	No	4	4
OLD13	Female	66	Senior High	No	4	4
OLD14	Female	86	Senior High	No	4	4
OLD15	Female	63	Senior High	No	4	4
OLD16	Female	62	College	No	5	5
OLD17	Female	64	College	No	4	4
OLD18	Male	69	Senior High	Yes	4	4
OLD19	Female	68	Senior High	No	4	5
OLD20	Female	66	College	No	5	4
OLD21	Female	66	Elementary	No	5	5
OLD22	Male	64	Senior High	No	4	5
OLD23	Male	66	Senior High	No	4	4
OLD24	Male	72	Senior High	No	5	4
OLD25	Male	68	Senior High	No	5	5
OLD26	Male	86	College	No	4	4
OLD27	Male	76	College	No	4	4
OLD28	Female	71	Elementary	No	3	3
OLD29	Female	72	College	No	5	4
OLD30	Female	69	Junior High	No	5	5
OLD31	Female	79	Elementary	No	5	3
OLD32	Female	73	Elementary	No	3	4
OLD33	Female	68	Senior High	No	4	4
OLD34	Female	67	Junior High	No	5	5
OLD35	Female	68	Elementary	No	5	4
OLD36	Female	65	College	No	5	5
OLD37	Male	74	College	Yes	5	5
OLD38	Female	64	College	Yes	5	5
OLD39	Female	78	Elementary	No	5	5

N3	Q1	Q2	Q3	F1	F2	F3	F4	F5
4	4	5	4	5	5	4	4	4
4	5	5	5	5	5	5	5	4
5	4	5	4	4	4	5	5	5
4	4	4	4	5	5	4	4	4
4	4	4	4	5	5	4	5	5
5	5	5	5	5	5	5	5	5
5	4	4	3	5	5	5	5	4
3	5	5	5	5	5	5	5	4
5	4	4	4	5	5	4	4	4
5	4	4	4	4	4	4	4	4
5	5	5	5	5	5	3	5	5
4	4	3	4	5	5	4	4	4
5	4	4	3	5	5	5	5	4
4	4	3	4	4	4	4	4	4
4	4	5	4	5	5	3	5	5
4	3	4	4	5	5	3	3	3
5	3	4	4	5	5	4	4	4
5	5	5	5	4	4	5	4	4
4	5	5	5	5	5	4	4	5
4	4	4	4	4	4	4	4	4
5	3	4	4	5	5	4	4	5
4	4	4	4	4	5	5	5	5
4	4	4	4	4	4	3	3	3
5	4	4	3	5	5	5	5	4
3	5	5	5	5	5	5	5	4
5	4	4	4	4	4	4	4	4
5	5	5	5	5	5	3	5	5
4	4	3	4	5	5	4	4	4
5	4	4	4	4	4	4	3	4
5	4	4	3	5	5	5	5	4
4	4	3	4	4	4	4	4	4
4	4	5	4	5	5	3	5	5
5	5	4	4	5	5	4	4	4
5	4	4	3	5	5	3	4	5
5	4	5	4	5	5	4	3	4
5	4	5	5	4	4	5	5	5
5	4	3	4	5	5	4	4	4
3	4	4	4	4	4	3	4	4
5	5	5	5	5	5	5	5	5
3	4	3	3	4	4	4	4	4
5	5	5	5	5	5	5	5	5
5	5	2	5	5	5	5	5	4
5	4	4	4	5	4	5	4	4
5	4	5	4	4	4	4	4	5
4	5	5	5	5	5	5	5	5
5	4	4	4	4	4	4	4	4
3	4	4	4	4	4	4	4	4

5	5	5	4	5	5	5	5	5
5	5	5	4	5	5	5	5	5
5	5	5	5	5	5	5	5	5
4	3	3	3	4	4	4	4	4
4	5	5	5	4	4	4	4	4
4	5	5	5	5	5	5	5	5
4	4	4	4	4	4	4	4	4
5	5	5	4	5	4	5	4	4
4	4	4	4	4	4	4	4	4
4	5	5	5	5	5	5	5	5
4	5	5	5	5	5	5	5	5
5	5	5	5	5	5	5	5	5
5	5	5	5	3	4	3	4	4
4	5	5	5	5	5	5	5	3
4	5	5	5	5	5	5	5	5
5	5	5	5	5	5	5	5	5
5	5	5	5	5	5	5	5	5
4	5	5	5	5	5	5	5	5
4	5	5	5	5	5	5	5	5
3	4	4	4	4	4	4	4	4
5	4	5	5	5	5	5	5	5
5	4	4	4	4	4	4	4	4
4	4	4	4	4	4	4	4	4
4	4	4	4	4	4	4	4	4
5	2	4	4	5	5	5	5	5
5	5	5	4	5	5	5	5	5
4	4	4	4	4	4	4	4	3
5	4	4	5	5	5	5	5	5
4	4	4	4	4	4	4	4	4
5	4	4	5	4	4	4	4	4
5	5	5	4	5	5	5	5	5

P1	P2	P3	EOU1	EOU2	EOU3	PU1	PU2	PU3
3	3	3	4	4	4	4	4	4
3	3	3	5	4	5	5	5	5
4	4	4	4	4	4	3	4	4
4	4	4	5	5	5	5	5	5
4	4	4	5	5	4	5	4	5
4	4	4	5	5	5	5	4	5
3	4	3	3	4	4	5	4	4
5	4	5	4	3	3	4	4	4
5	5	5	4	5	5	5	5	5
4	3	4	4	4	4	3	3	3
5	5	5	5	5	5	5	5	5
3	3	4	4	4	4	5	4	4
3	4	4	4	5	4	4	4	4
4	4	4	4	4	4	4	4	4
3	4	4	4	4	4	4	4	4
4	4	3	3	3	4	4	4	4
3	3	3	4	4	4	4	4	4
3	3	3	5	4	5	5	5	5
5	4	4	4	4	4	4	4	4
3	2	2	3	4	3	4	4	4
4	4	4	4	3	4	5	4	4
4	4	4	5	5	5	5	4	4
2	2	2	4	2	2	4	4	4
3	4	3	3	4	4	5	4	4
5	4	5	4	3	3	4	4	4
4	3	4	4	4	4	3	3	3
5	5	5	5	5	5	5	5	5
3	3	4	4	4	4	5	4	4
3	3	3	3	3	3	4	4	4
3	4	4	4	5	4	4	4	4
5	5	5	4	4	4	4	4	4
3	4	4	4	4	4	4	4	4
3	3	3	4	4	4	4	4	4
3	3	3	5	4	5	5	5	5
3	3	3	4	4	3	3	4	4
4	4	4	5	5	5	5	5	5
5	4	5	4	3	3	4	4	4
4	3	4	4	4	4	3	3	3
4	4	4	4	4	4	5	4	4
3	3	3	3	3	3	5	5	5
5	5	5	5	4	5	5	5	5
3	3	3	4	5	5	5	5	5
3	3	4	5	4	5	5	4	4
4	4	5	5	4	5	5	5	5
5	5	5	5	5	5	5	5	5
4	4	4	5	4	4	4	4	4
4	4	4	4	4	4	4	5	4

3	4	4	5	5	5	5	5	5
4	4	4	5	4	5	5	4	4
5	5	5	5	5	5	5	5	5
4	4	4	3	3	3	4	4	4
4	4	4	4	4	4	4	4	4
5	5	5	5	5	5	5	5	5
2	3	3	4	3	4	4	4	4
5	4	4	5	5	5	5	5	5
3	3	3	4	4	4	4	4	4
4	4	4	4	4	4	5	5	5
5	5	5	5	4	4	5	5	5
4	3	3	5	5	5	4	4	4
4	4	4	3	3	2	4	4	4
5	5	5	3	5	5	2	5	5
4	4	4	5	5	5	5	5	4
5	5	4	5	5	5	5	5	5
5	4	4	4	4	4	5	5	5
5	5	5	5	3	5	5	5	5
5	5	5	5	5	5	5	5	5
4	4	4	4	4	4	4	4	4
4	5	5	5	5	5	5	5	5
4	4	4	4	4	4	4	4	4
4	3	4	4	5	4	4	4	4
4	4	4	4	4	4	4	4	5
4	4	4	4	4	4	4	5	5
5	5	5	5	5	5	5	5	5
4	5	5	5	5	5	5	5	5
5	5	5	5	5	5	5	5	5
4	4	4	4	4	4	4	4	4
4	4	5	4	4	5	4	5	4
3	3	5	3	5	3	5	5	5

BI1	BI2	BI3
4	4	4
4	5	4
4	4	5
4	4	4
4	4	5
5	5	5
4	4	4
5	5	4
4	4	4
4	3	4
5	5	4
4	4	4
4	4	4
2	4	2
4	4	4
4	3	3
4	5	5
5	5	5
4	4	4
4	4	4
4	4	4
4	4	4
2	4	3
4	4	4
5	5	4
4	3	4
5	5	4
4	4	4
3	3	3
4	4	4
2	4	2
4	4	4
4	4	3
4	4	4
4	4	4
5	5	4
4	3	3
2	2	2
5	4	4
4	4	4
4	4	4
4	5	5
5	5	5
4	4	4
5	5	5
4	4	4
4	4	4

5	5	5
4	4	4
5	5	5
4	4	4
3	3	3
3	3	4
4	4	4
5	5	5
5	5	5
3	3	3
4	4	4
4	5	5
5	5	5
5	5	5
3	3	3
5	5	5
4	4	4
4	4	5
5	5	5
4	4	4
5	5	5
4	4	4
4	4	4
4	4	4
4	5	5
4	4	4
5	5	4
5	5	5
4	4	4
5	5	4
5	5	5
